# Anatomy, Physiology, and Pathology

**Published:** 1837-01

**Authors:** 


### PART THIRD.

### Selections from the Foreign Journals.

---

### ANATOMY, PHYSIOLOGY, PATHOLOGY.

### *On the Theory of Congestion, Hemorrhage, Plethora, and Inflammation.* By Professor NAUMANN, of Bonn.

[WITHOUT pretending to assent to all the views maintained in the following essay, we think it well deserving the attention of our readers, for its own sake, and still more as showing the present opinions of one of the most enlightened physiologists in Germany on the momentous subjects of which it treats.]

I. CONGESTION. When the function of a part increases in activity, its capillary vessels are observed to receive a more ample supply of blood. During the process of digestion, the pale redness of the mucous membrane lining the stomach becomes exchanged for an intensely red colour. This is most conspicuous in children, and least so in old persons. In new-born children, the mucous membrane of the stomach is at all times distinguished by an intenser degree of redness. Digestion being concluded, the increased redness gradually disappears. If the digestive function be too powerfully excited, the repletion of the capillaries becomes still more strongly marked, and continues for a longer period. This phenomenon corresponds to that state wherein the stomach is vulgarly said to be "more sensibly felt." In a precisely similar manner the eye is affected when its functional activity is over excited. The same phenomenon is witnessed with regard to the skin, as a consequence of friction or of the influence of very heated air. It is a fact, then, that the capillary vessels of a part become replenished with blood in the exact ratio in which the nerves of that part have become conductors of *peripheric impulses.**

When the degree of excitement is over-powerful, or its cause has become a permanent one, the peripheric impulses begin to overbalance the normal equilibrium of sensation; the capillary vessels of the part become more and more distended with blood, and a quantity of the latter fluid, incompatible with health, encumbers the organ. From the earliest period this state has been denominated *symphoresis* or *congestion*. At the present day we speak of "simple vascular irritation," which is likewise termed "active congestion," and is recognized by the following signs: At the seat of congestion increased warmth, together with a sense of pressure or tension, are felt, and the function of the part is somewhat impeded. When these symptoms give way, augmented secretions usually follow, from the organ itself, if it be a secreting one; if not, from one or more of the general organs of secretion, especially from the skin, the kidneys, or the intestines. Well-defined symptoms of active congestion, accompanied by copious though not morbidly augmented secretions, represent that state which has been denominated "turgor vitalis," the phenomena of which are most easily observed on the cutis. Since organs in such a condition (though appearing to contain an excess of blood,) perform their functions with more than common energy; since, moreover, all morbid sensation is absent, this turgor can only be regarded as an attribute of health. There are

* By the term "*peripherical nervous impulse*," the author means to express the influence which is transmitted from the nerves of any particular part of the body to the centres of the nervous system, i. e. the brain and the spinal cord. "*Central nervous impulse*," on the contrary, is meant by him to designate the influence which is conveyed back again from those centres to the nerves of a particular part.—TR.

however, other conditions which lie midway between the above one and active congestion.

When an organ has become the seat of permanent congestion, the symptoms are particularized by a peculiar degree of vacillation; now augmenting, now decreasing; at times unexpectedly vanishing altogether, and then as suddenly reappearing. The more frequently an organ has been affected with congestion, the more easily it is again called forth. If the congestion be very strongly pronounced, the whole circulating apparatus is called upon to participate in its effects; the heart's contractions become more vigorous, the pulse more frequent, full, and developed. Again, if the seat of the congestion be in one of the more important organs, the pulse is rendered still more frequent, perhaps fuller, but always somewhat hard and labouring; the latter being always in proportion to the degree of functional excitement in the affected part. Evanescent congestive phenomena, which manifest themselves now in this, now in that organ, then again in several organs at once, constitute what we understand by the term "orgasm."

The process of active congestion is usually explained as follows: When a part is over-excited, its vitality, if not actually increased, at least becomes quickened. Hence augmented heat; and, as on the latter depends the more rapid consumption of the fluids, an increased afflux of blood is thus called for, which may end in an accumulation in the part affected. It was in this sense that Hippocrates explained congestion.* Essentially agreeing with the above is another theory of congestion, according to which a local excess is assumed in the vegetative or formative process, which demands a more ample supply of vivifying matter, and consequently an accelerated afflux of blood.

That acute pathologist, Stieglitz, has of late years protested against this theory; and, in truth, it is open to numerous objections. If the arteries do convey an increased quantity of blood to a part, wherefore should not the veins carry it away again in an equal proportion? But, if such were the fact, the circulation must be necessarily more rapid at the seat of congestion than in the rest of the body. Even though we granted this to be the case, it would still be impossible therewith to reconcile the phenomena of an augmented *vis formativa* in the part; for experience shows us in every instance where the general circulation is preternaturally accelerated, (thus, in all febrile diseases,) a decrease in the vegetative process, even to emaciation. The question is, however, first to be answered, by the exercise of what power can an individual organ create for itself an increased afflux of blood? The "vis suctionis" or "attractionis" of certain pathologists rests on no better foundation than that of hypothetical fiction; and the assertion is equally gratuitous that, when the sensibility of a part is increased, an interrupted equilibrium of polarity† occasions an augmented afflux of blood. If the cause be referred to a greater irritability or contractile power in the vessels themselves, we must object that those properties are entirely wanting in the capillary vessels, and that the replenishing of the arteries is exclusively dependent upon impulse from the heart. As, however, with every contraction of the left ventricle, the blood is impelled with equal force in every direction and in all the arteries, it becomes an impossibility that congestion in a single part should arise out of a general quickening of the circulation from the central organ. And, even if it could thus originate, an *accumulation* of blood could still not be thereby occasioned, since the blood thus conveyed to the part by accelerated motion would then with equal rapidity be again carried away from the organ.

It remains for us, in our enquiry into the pathogenesis of congestion, to consider the mutual relations betwixt the blood and the nervous medulla. So long as this mutual relation continues in a normal state, no congestion takes place. A morbid predominance of the central nervous influence, (wherein the nerves perform the

* "Caro sauciata subresiccatur et subcalescit, et humiditatem in se ipsam a vicinis tum venis tum carnibus trahit; ubi autem attraxerit, intumescit (et inflammatur) et dolorem exhibet."—*De Morb.* lib. i. cap. 2.

† Kreissig and certain other pathologists are wont to compare the blood and the nervous medulla to the two poles of electricity.—Tr.

part of conductors from the centre to the periphery,) cannot under any circumstances originate *à centro.* For, according to the laws of organic unity, the central impulse towards individual organs is augmented only *in consequence* of previous excitement to that effect; that is to say, in consequence of the nerves having first conveyed an impression from the periphery to the centre. But, even supposing an impulse affecting an individual organ to originate directly *à centro,* the only result to such organ would be increased nutrition, and therefore renewed energy of its functions. It is far otherwise with regard to the predominance of peripheric nervous influence; for, in proportion as the nerves act as conductors *away from* the capillary vessels, the blood contained in the latter becomes deprived of a portion of the nervous influence indispensable to it. The genuine nutritive or formative process thus proceeds more slowly; the blood flows back in diminished quantity, and therefore accumulates in the capillary vessels, there assuming properties of an irritative nature, proportioned to the deficiency of nervous influence. In a word, congestion (hyperæmia) establishes itself. In a measure corresponding with the importance and the size of the organ itself, this morbid state must sooner or later react upon the whole circulation.

The relation of a part to the whole will be greatly elucidated by a glance at the so-termed "passive congestion," which has been likewise called "torpid stagnation of the blood." The latter state is observed solely in organs whose power of resistance has been greatly exhausted, and is particularly liable to arise out of frequent relapses, or out of habitual active congestion. Unquestionably, a constant irritation of the extreme peripherical nerves of one part must ultimately blunt their powers of reaction. The conducting powers of those nerves will become diminished, and thus the equalizing impulse from the centre will be rendered more labouring and less perfect. The capillary vessels of an organ thus abandoned will gradually grow more and more distended, and a permanent state of repletion with blood must ensue.

The phenomenon next to be considered is the morbid distention of the veins which we observe at the seat of congestion. In these vessels circulation is not entirely suspended, though it becomes more and more inert from the arteries conveying to the organ an excess of blood, in relation to its depressed vitality. Since, then, the blood arrives in a very slow course through the intermediate capillaries into the veins, these latter participate less and less in any impulse from the heart. The blood thus accumulates in and distends those torpid, unresisting channels, through which its progression can only be effected by a mechanical repletion or a species of overflowing, so to term it. By this lingering of the blood in the veins, however, the depletion of the capillaries must necessarily be greatly impeded. Hence nutrition rapidly declines in the part, although the latter may be absolutely gorged with blood. At the same time, in proportion to the degree of loss of power in the nutritive process, fluids of a lesser degree of vitality, or even with properties altogether foreign to the part, will accumulate in the interstices.

Passive congestion occurs most readily in organs which, notwithstanding a perfect connexion between their arteries and veins, at the same time possess few or no nerves; for in such organs the blood only derives a portion of nervous influence indirectly through the anastomoses of the capillary vessels.

II. Hemorrhage. With congestion, hemorrhage is intimately connected. The latter may, with equal propriety, be divided into active and passive.

1. *Hemorrhage with Irritation.* This commonly occurs after active congestion has become more or less habitual, without, however, having yet assumed the character of passive congestion. Organs in such a state always evince more or less of irritation, whilst their capillary vessels are day by day becoming more relaxed. The hemorrhage of which we now speak proceeds exclusively from mucous membranes, therefore from secreting organs. Such surfaces are liable to be directly acted upon by the product of secretion itself, or by influences, more strictly speaking, external; as, for instance, by the atmospheric air. By means of such influences, the already enfeebled energy of central nervous impulse is still more forcibly counteracted.

Nervous influence is thus gradually withdrawn from the blood circulating through the capillary vessels, and that fluid becomes consequently much more susceptible of the influence of the morbific stimuli from without. It is, however, evident that the normal process of organic secretion from the capillaries must decline the moment that the character of the fluid those vessels contain can be determined according to the law of exosmosis and endosmosis. The albuminous and fibrinous contents of the blood will be more tenaciously retained by the latter. Hence the coagulable deposit from the blood, that which affords materials for the formation of the capillary vessels, will (for reasons hereafter to be developed,) cease to take place, at least in a perfect manner. The blood must thus escape from its confinement; in other words, capillary hemorrhage must ensue. From the connected state of the capillary system, vessels whose channels are still unimpaired are, in their turn, relieved of their burden by means of the hemorrhage. By this very act, the predominance of peripheric nervous influence is undermined, and central nervous impulse, no longer meeting with the same opposition, gradually resumes its sway. Equilibrium being thus restored between the blood and the nervous medulla, the albuminous deposit is again formed, new capillary vessels organize themselves in place of those which were destroyed, and the hemorrhage ceases of its own accord.

2. *Hemorrhage attended by Exhaustion.* When this occurs, the capillary vessels of the part are in a very high degree distended, and the veins greatly enlarged. The nerves are now scarcely capable of performing their functions of conductors from the centres, and, upon the whole, their conducting power is very inconsiderable; in a word, the vital energy of the organ has become greatly impaired. Here the hemorrhage must necessarily be profuse, because the capillary vessels within a large circumference participate in it. The blood which issues forth is almost always dark coloured, since a great portion of it proceeds from the distended veins. The albuminous deposit takes place slowly from the arterial blood, which latter is probably itself of a degenerate quality. For these reasons, a longer period elapses ere the capillary system has recovered its continuity, and, on the slightest occasion being given, the hemorrhage returns. It is evident that, so long as the capillary vessels maintain a state of perfect integrity, no capillary hemorrhage can occur; for not a single globule of the blood can by any chance escape through the parietes of those vessels.

III. Plethora. In approaching the question of plethora (Polyæmia,) we find the received ideas on this head likewise subject to many doubts. How is it possible for true plethora to arise? Granting an excess of chyle to be superadded to the sanguineous mass, the latter must have been drawn upon to no slight amount to produce this. For the due elaboration of the chyme, and the separation from it of the chyle, an augmented secretion of saliva, of bile, of the pancreatic juice, &c. is rigidly demanded. What, therefore, the blood would thus gain on the one hand, it would have already lost on the other, since the first acts of hæmatosis can not be imagined to occur without functional excitement of the gastric organs. An absolute excess of blood can, then, scarcely be produced by such means; for, even supposing that more chyle reached the mass of blood than the loss in digested fluids amounted to, the assimilation of such chyle would be proportionately less perfect. The plethora of children proves that in such case the excess would weigh less upon the blood-vessels; that it would rather seek to dispose of itself in nutrition, through the medium of the capillaries. Here, however, being ill adapted for the normal formative process, it could only serve to overload and distend the lymphatic system.

With a view to reconcile the phenomena attendant on plethora with physiology, we may recognize the following forms:

1. *Plethora arteriosa, or Erythrosis.* The blood is here rich in fibrine and in bright red pigment; it coagulates very rapidly, and has an intensely red colour. The capillary system is very strongly developed, as regards space; the complexion is florid. The blood circulates rapidly through the capillary vessels; the renovation of organized matter (nutrition) proceeds vigorously; for which reason the colouring matter becomes proportionably less black during its progress through the capillaries. This state of things in some measure corresponds with what has been termed the *arterial constitution*, although the latter may be regarded as the one which deviates the least from perfection, as is proved by the great functional capacity of the lungs in those who possess that constitution. So soon as external influences call for an extraordinary reaction of central nervous impulse, the irritative properties of the blood easily acquire the preponderance over the resisting powers of the nervous system. The consequences of this are the phenomena of congestion in several organs at once, more particularly in the lungs and in the brain. In proportion as the frequency and the extent of such congestions increases, the metamorphosis of matter,—that is to say, the true formative process,—must meet with more and more obstacles. Thus may excess in the quantity of the blood in reality occur. As, however, with the increasing accumulation of blood at the origin of the veins the atmospheric influence over that fluid must diminish, erythrosis at length usually degenerates into the next variety of plethora.

2. *Plethora venosa, or Cyanosis.* Here the fibrinous contents of the blood are comparatively far less considerable than its albuminous ones. The blood does not so speedily coagulate, but its colour is intensely red. The complexion is distinguished rather by a blue red than by a bright red colour. The blood is less adapted for the formative process; nutrition and the metamorphosis of substance are more languid than in the former variety. The phenomena of congestion and of orgasm arise the more readily, especially in the abdominal organs. For in these cases nature exerts herself to purify the blood by an extraordinary secretion of bile. Thus the liver, the spleen, and the organs connected with the whole system of the vena portæ, maintain a great degree of functional excitement; a condition so favorable to the development of congestion.

3. *Plethora nervosa.* Here the actual quantity of blood may be smaller than in the state of health; but the nervous system is possessed of so little energy, and its peripheric impulse is so potent, that, on the slightest occasion being afforded, even the normal stimulus of the blood becomes a disturbing agent. This species of irregularity is most frequent in persons of the female sex. Individuals thus affected are commonly distinguished by a vivid, though at the same time delicate, redness of the complexion, which colour however frequently alternates with paleness. In such persons mental emotions are particularly apt to be succeeded by the most violent orgasm, which, by repetition, at length gives rise to permanent congestions. These make frequent and sudden transitions from the uterine system to the thoracic organs, from thence to the brain, and back again.

4. *Plethora vera.* Of this we can distinguish two forms or varieties: *a. Plethora anatomica.* The removal of large members by amputation is apt to be succeeded by true plethora. The process of sanguification has undergone no impairment: hence the same quantity of blood is generated as before, but the space is no longer adequate for its lodgment. A similar result may exhibit itself where a large portion of blood, which had for a long period been excluded from the circulation, is again suddenly restored to the latter; as, for instance, when the spleen rapidly collapses subsequently to great and protracted enlargement of that organ.—*b. Plethora hæmato-poietica.* This form has been till now little attended to, and appears to belong peculiarly to the female sex. The process of sanguification is here more than usually active, whilst, on the contrary, the formative process,—i. e. the nutrition of parts and the natural secretions,—are both tardy and imperfect. There is a great disposition to hemorrhage, especially from the genital organs, the lungs, and the intestinal canal. Copious and repeated hemorrhage is not attended with a proportionate effect in reducing the powers, nor does it exercise any remarkable influence over the natural properties of the blood itself.

IV. INFLAMMATION. It is well known that the phenomena of inflammation may be studied with the greatest degree of accuracy, when artificially provoked in healthy animal tissues endowed with vitality, and which admit, at the same time, of very narrow investigation; as, for instance, in the natatory web of frogs. Here then the following circumstances are of moment. When a powerful and searching irritant begins to exert its sway, we quickly perceive a greater number of globules in the capillary vessels of the part than the moment before. After a short period, we observe an augmented repletion of these vessels with blood, and consequently a distention thereof. In proportion as this becomes more and more striking, a number of minute capillary vessels, which were not previously witnessed, are seen to develope themselves, and to become replete with red blood. This apparent local development of the capillary system extends in every direction, so that the irritation, in relation to its focus, may be said to radiate from a centre to a circumference. A greater number of globules is compressed together within the capillary vessels, and thus minute channels, containing red blood, everywhere become manifest. The phenomena of active congestion are thus established; the circulation throughout the body is at the same time accelerated. But we must guard ourselves against considering this accelerated movement of the blood as the cause of the local phenomena hitherto described: for, at the seat of incipient inflammation (i.e. in the capillaries,) the motion of the blood cannot have become accelerated, otherwise the accumulation of globules therein could not take place. We have been too long deceived by this concomitant acceleration of the general circulation. The cause of this accompaniment is to be sought for in the effect produced on the general sensibility (the *cœnæsthesis,)* which is inseparable from the experiment, and the result of which necessarily is, that the contractions of the heart succeed each other more rapidly than before. If the frog's heart be previously extirpated, and the great blood-vessels tied, such acceleration of the blood's motion is not observed. But it is superfluous thus to extend the experiment; for the congregating of the globules could still not be explained by accelerated circulation: were the course of the blood at the part threatened with inflammation really accelerated, the respective interspaces between the globules would not be lessened; they would only then glide at shorter intervals over the field of vision: thus their movement alone would be quickened, the interspaces remaining the same as before. Accelerated motion of the blood could never effect augmented repletion and distention of the vessels; otherwise, the same cause in healthy persons must, in like manner, produce inflammation, or, at any rate, congestion. In the latter instances, however, every part of the body receives its due proportion of blood, which only passes through it more rapidly, owing to the more vigorous impulse from the heart. The crowding together of the globules in the capillary vessels, together with the distention and the reddening of the latter, can therefore have no other cause than in an impediment to the reflux of the blood from these vessels.

If, in experimenting as above on the natatory web of the frog, we make use of very powerful local irritation, the transflux of the blood is for a time totally impeded, and that fluid for an instant assumes a retrograde movement; after which, its progressive motion is restored with a degree of impetus. If the irritative stimulus be very violent, this resumption of the progressive movement is, in its turn, instantly followed by an entire stagnation of blood in the capillary vessels. The very same phenomenon is produced by a comparatively very slight irritative stimulus, when the vital energy of the tissue operated upon is greatly reduced. The circulation is again equalized, provided the vis a tergo of the arteries is sufficiently powerful to overcome the impediment.

When inflammation is once established, the globules of the blood appear crowded together and motionless in the greatly distended capillary vessels, which must therefore have become impermeable. Inflammation, then, developes itself from the motion of the blood in the capillaries becoming more and more tardy, in consequence of congestion from the increasing accumulation of blood in those vessels, from the more irritative properties with which such blood becomes endowed. At the seat of congestion, circulation still goes on; at the seat of inflammation it is at an end.

The physiological rationale of the development of inflammation is, then, briefly as follows: the blood accumulating gradually in the capillary vessels, owing to the congestion in a part, becomes more and more a source of abnormal irritation to the nervous ramifications. Peripheric nervous influence is thus rendered predominant. On the other hand, the central nervous influence becomes restrained, and for this very reason the organic power of resistance or reaction of the inflamed part is lessened. The blood becomes deprived of the nervous influence indispensable to it, and the healthy renovation of substance must be almost totally interrupted.* The globules of the blood become more and more crowded together. Owing to the impeded motion of the blood, its fibrine (from its organic affinity to the cruor,) will be most easily held back, whilst the rarer parts of the fluid still find their way into the venous stream. When the increasing stagnation ceases to admit even of this, the serum of the blood at length penetrates the coats of the capillary vessels, and collects itself within the cellular texture, in the shape of what has been called puriform serum. At the same time the fibrine and the globules are retained in close conjunction with each other in the capillary vessels.

So long as the state of simple congestion continues, the fibrine alone is more closely retained by the blood; for its affinity to the red pigment increases in the same proportion as the exercise of nervous influence over the blood becomes diminished. Hence nutrition is rendered more imperfect. As we have already seen, (where the blood is already poor in albuminous and fibrinous matter,) this may be carried to such a point that a plastic deposit is no longer formed by the blood, and that capillary hemorrhage therefore becomes inevitable. When the point of inflammation is reached, a very great excess of albuminous and fibrinous matter must accumulate at the focus of the inflammation. The power, therefore, of the blood in those parts will be the sooner exhausted, and plastic deposits will take place within the capillary vessels themselves; whilst, on the other hand, the remaining serum will be the more actively pressed out of these vessels. The blending together of the globules with the fibrine of the blood is attended with a partial destruction of the former. Hence, the serum occupying the interstices of the cellular tissue is usually tinged with red. The sero-sanguineous infiltration is owing to the partial solution of the envelopes of the globules in lymph; those envelopes constituting the pigmentum. In the parts surrounding the focus of the inflammation, the blood will have acquired much of an irritative property, without partaking of the entire stagnation in its vicinity. This blood will be transmitted to the general circulation, and may impregnate the whole mass of blood with an irritative principle. It is upon this ground that we must account for the phenomenon of symptomatic fever.

Respecting the products of inflammation, I shall be silent. A little investigation will show that the views communicated in the above essay agree with the phenomena of which those views are intended to be explicatory. Of a too vigorous afflux of blood as the proximate cause of inflammation, there can scarcely now be any question.

*Rust's Magazin für die gesammte Heilkunde, 45tes Bandes, 3tes Heft.*

---

*On Gastroperiodynia.* By THOS. A. WISE, M. D.

As I have not seen any account, by European physicians, of a peculiar disease known in this country by the generic term *sool*, I am induced to offer a few remarks on it, in hopes of drawing the attention of the profession to the subject. Gastroperiodynia, or periodical pain in the stomach, often commences with symptoms of indigestion and heartburn, succeeded by a feeling of uneasiness in the scrobiculus cordis, slight and occurring at irregular intervals at first, and lessening gradually while the pain increases in violence. In severer cases, the paroxysm of acute pain generally occurs rather suddenly, when the stomach is empty; sometimes early in the morning, or three or four hours after dinner; increasing slowly when the digestive process is finished, and the activity of abdominal organs has ceased. The excruciating pain is confined to the pit of the stomach, particularly towards the right hypochondrium, although the neighbouring parts often participate, as the muscles of the loins, &c. The pain is of a cutting or gnawing nature; and during the paroxysm is intense, from which the patient generally obtains temporary relief

* The same effects must be produced on organs which are not themselves possessed of nerves; since to those a nervous influence is imparted from neighbouring organs through the medium of the anastomosis in the capillary system.

from pressure. For this purpose he often turns upon his breast and places a hard ball in the pit of the stomach, on which he rests his body, and keeps rolling from side to side. A few days since I ordered an unfortunate person, in this position, to turn round for me to examine him; but such was the agony, that he could not remain without the soothing influence of pressure. As a substitute, he drew from under his clothes a piece of prepared bamboo, an inch in diameter, and a cubit in length, round at one end. This extremity he placed to the pit of the stomach while he rested the other upon the floor; and by bending the body over it, produced such a degree of violent pressure, that at first I feared he would injure himself, and even penetrate the abdomen. He continued in this position while I gained the necessary information of his state, and then returned to his former position, commenced rolling from side to side upon the hard ball, placed in the pit of the stomach, and his low wailing proved how intense were his sufferings and wretchedness. This was an acute form of the disease, and until checked by remedies, it occurred daily about four o'clock, and he continued in dreadful agony the whole night. These paroxysms usually occur at intervals of a day or two, and remain from two hours to as many days, with occasional intervals of ease. During these accessions the state of the pulse, although full, continues slow and natural, and there is not usually more heat of body than is occasioned by the continual movement. In the intermissions the patient feels well, and all the functions are performed as when in perfect health. The tongue is clean, but sometimes parched, appetite good, and alvine secretions healthy and regular. So painful are the paroxysms of this form of the disease, that in the Sanscrit works on medicines it is supposed to be produced by the deadly weapon in the hands of Siva, the destroying power of the *triad*; and so incurable that when it attacks an individual, it is declared that even Siva himself cannot remove it. As a natural conclusion to such reasoning, those afflicted add, what use is there in requesting the assistance of a physician? It frequently happens that the unfortunate individual, in despair of being cured, commits suicide, as the only means left of relieving himself, and of propitiating the anger of the Deity.

I have not been able to obtain any certain evidence of the causes which produce this disease. It appears to be the effect of a combination, individually slight; but when occurring in a peculiar constitution, produces this distressing complaint. I have generally known it to occur in the young and robust male; but without being produced by any particular exposure to the weather, or owing to the quantity or quality of the food. The accession seems oftener to occur when the organs are in a state of inactivity, than from causes increasing the nervous susceptibility. I have not seen it in females, although there is no reason why they should not be also affected; but those of a nervous temperament in general are not so liable to it as might be supposed. The severe pain which occurs in paroxysms, the relief obtained by pressure, and the absence of participation in the circulating system, indicates, with sufficient accuracy, the nervous nature of the disease.

The resemblance of paroxysms of gastroperiodynia to those of tic-douleureux is marked, not only in the nature and severity of the pain, but in the means of relief and the difficulty of curing both diseases. The severe paroxysms of otalgia which so frequently occur in this country, some forms of asthma, colic, &c. form examples of the same species, varying more from the situation and nature of the parts affected, than from any difference in the disease itself. The diet I have found most efficacious in gastroperiodynia is liquid farinaceous food, with boiled milk, which in some cases I have seen diminish the violence of the paroxysms. In strong and plethoric persons blood should be taken from the arm; and may sometimes be repeated generally or locally with advantage. During the paroxysms, pressure, heat, tinctures, ether, peppermint, anodynes, particularly opium, henbane and camphor, are of use in diminishing the severity of the pain. During the intervals the oxyd of zinc and bismuth, carbonate of iron, sulphate of quinine, are all in some cases of use; but it must be acknowledged that the relief is often of a temporary nature; and in some cases diminish, in effect, or even seem to aggravate and prolong the succeeding paroxysm.—*India Journal of Medical Science, March* 1, 1836.

*On the Oxide of Bismuth in Yellow Fever.* By J. MAGRATH, Esq.

BELIEVING that I have met with a medicine capable of exerting a decidedly beneficial influence in that stage of yellow fever, in which every remedy hitherto used has either totally failed, or at the most has been so very precarious in its effect, as to leave it doubtful whether the patient was indebted to the means employed or to nature alone for the relief obtained, I beg leave to make it known. The remedy I allude to is the oxide of bismuth, and the period of the complaint, the latter part of the first or the commencement of the second stage of the fever, in which sub-acute inflammation of the mucous membrane of the stomach exists, which I believe is always the case previous to the appearance of black vomit. I have successfully employed the remedy in two instances after this last symptom has set in; nevertheless, I fear it will seldom be of use when the discharge of that fluid is at all considerable, as then it is to be apprehended that too great disorganization has taken place; but where inflammatory action has already been actively combated by general and if necessary topical bloodletting, by the use of mercurials, a blister, &c., I cannot assert with confidence that it will, in many instances, save the life of the patients, when none of the other remedies hitherto employed have any chance of succeeding. I have generally been in the habit of combining the carbonate of soda with the bismuth, and giving them in doses of three grains each every second hour as long as I saw an indication for their continuation.—*Jamaica Physical Journal, March and April*, 1836.

---

*On the Cachexia Africanorum.* By JOHN FERGUSON, M. D.

THE characteristic symptoms are given to the Cachexia Africanorum either by the deficiency or by the vitiated state of the blood, though I have seen it retain its inflammatory or pyrexial character to the last. The strength is lost; the muscles are soft, the expression of the countenance is dull, and heavy, and desponding, the colour of the skin changes from a deep black to a yellowish brown; it loses its gloss, is dry and cold and flaccid; the nails are white; the hair becomes lighter in colour and dry; the inside of the lips, the gums, the tongue, the palate and pharynx are more or less deprived of their colour, sometimes indeed they are colourless; the palms of the hands are white and dry; the backs of the hands are swollen; the carotids are seen beating violently, and the jugulars are generally pulsating; the heart, even when the body is at rest, sometimes beats with inordinate force and frequency; and, on the least exertion, it assumes a jerking action and strikes forcibly against the chest, and gives out a bellows' sound; the pulse at the same time has a sharp jerking beat and is much accelerated. Walking a few paces brings on dyspnœa or anxiety, a palpitation of the heart that is visible all over the chest and in the epigastrium: rapid and strong pulsation in the carotids; and if the exertion be continued, the dyspnœa is fearfully increased, and a state resembling asphyxia eventually ensues. The eyelids are swollen in the morning and the countenance acquires a transparent appearance. At the close of the disease effusion takes place in the cellular tissue of the skin and in the cavities. There is often considerable œdema of the face from an early period. The belly is tumid, the appetite may be voracious or entirely lost, the digestion is greatly impaired and the motions are light coloured and sometimes earthy. There is often an irresistible desire to eat all kinds of earthy matter, such as decayed limestone, marl, the plaster on walls, and when these cannot be had, the dust on the floor, or the common earth on the ground. Calcareous earth is preferred, and I recollect seeing a considerable excavation on the side of a hill along which passed the public road, where the negroes on the neighbouring properties were in the habit of supplying themselves with an absorbent earth to allay the cravings engendered by the cachexia, or perhaps by other gastric disorders. The blood drawn from a vein looks like muddy claret and water, and when it has stood for some time exposed to the air, the coagulum is found to be loose and of a very dark colour, as if the oxygen of the air did not exert its usual effect upon it.—*Jamaica Physical Journal, January and February*, 1836.

*On Milk-Sickness.* By Drs. SHELTON, WHITE, and MACANELLY.

[IN the Transylvania Journal, for April, 1836, there are copious extracts from three inaugural dissertations, by Drs. Shelton, White, and Macanelly, giving an account of a disease having this name, and which is endemic in the western states of Alabama, Indiana, and Kentucky. It affects both man and beast, but chiefly cattle. It is very imperfectly described in the extracts, probably because familiar to the ordinary readers of the Journal, but many particulars respecting its localities and supposed causes, which cannot fail to interest the medical topographer, are given. The first two extracts give us rather an *idea* of the nature of the disease than a description of it. It is commonly attributed, in cattle, to something eaten or drunken by them; and, in man, to the eating of the flesh of animals who had been affected with the disease.]

"The disease is most severe during the driest season of the year, be that early or late. In a wet season we see but little of it. But one case occurred within our knowledge in the course of the last summer, which was brought on by eating dried beef that had been slaughtered the preceding fall. During that fall some twenty or thirty cases occurred in our county. The season was one of extreme drought, and the cases were most numerous in October and November, when it was at its height. I was called to a woman who had laboured under the disease for about twelve hours. She was found to be quite insensible to all external objects, extremely restless and anxious, pulse about one hundred in the minute, small and weak. She was harassed with perpetual retchings, countenance flushed, and eyes suffused and glassy; bowels constipated, extremities cold. Bleeding was attempted, but no blood could be obtained. Calomel was repeatedly given without effect, and she sunk in a few hours. Her husband was labouring under a milder grade of the same disease, but recovered in a few days. The above case is the only one in my practice in which the brain has been observed to suffer."

"We confess our ignorance of the true cause, but, whatever it may be, the stomach and duodenum appear to be the arena of its action; the liver refuses to furnish its accustomed secretion, and the sympathetic nerves are materially deranged."

"An animal affected with this disease is suddenly seized with rigors, and the whole frame becomes violently agitated; they lose the use of their limbs, and lie helpless in this state of universal tremor, until they expire. It is from this symptom, which is so apparent, that the disease has received the name of *trembles*.

"This disease has not occurred generally in every part of the West, but is confined to particular localities more or less limited. It frequently happens that the inhabitants of two or three adjoining farms will be subject to its attacks, while the surrounding neighbourhood remains entirely exempt. It sometimes takes a wider range, and some of the fairest portions of the West, in consequence of the prevalence of this loathsome disease, must ever remain an uninhabitable waste, unless the cause and remedy can be discovered."

One of the circumstances at once most interesting and most remarkable relating to this disease, is the strict limitation of its course to certain localities.

"In Blount county, Tennessee, there is a locality embracing not more than ten or fifteen acres, on which the disease has been known to originate for nearly, perhaps quite forty years. I was informed by the proprietor of the land that, by watching his cattle for a few years, he was enabled to discover the limits of the poison, and that, by enclosing the infected region in a fence, so as to exclude his live stock from it, the disease ceased to be troublesome, except when an animal got by accident into the enclosure. Another locality exists in the same State, Monroe county, about the size of the first, in which the disease, previously very annoying, was suppressed for eight or nine years by enclosing the seat of the poison. But, during the last few years, the enclosure having decayed and fallen down, so as to admit the ingress of cattle, the disease has again shown itself, and rarely fails to occur in animals that feed long on the spot.

"A third site occurs on the Chattahooche river, in Georgia. It is an elevated piece of bottom land, containing scarcely more than five acres, shut in by the river and by bluffs on every side. This spot was easily discovered to be the source of the disease, and was accordingly fenced in securely by the owner, and the evil thus effectually removed."

The following extract would seem, contrary to the common opinion which attributes the disease to the ingestion of poisonous plants, to trace it distinctly to the water drunken by them. It is much to be regretted that no chemical analysis of the suspected springs has been made.

"A respectable farmer informed me, that his cattle had been in the habit of frequenting a pasture ground in company with his neighbour's, on the opposite side of a creek from him. In returning home, his cattle were obliged to cross the creek. For many years not a case of the disease appeared among them, whilst his neighbour lost some forty or fifty head during that time. The animals of the latter did not cross the creek, but drank at another stream. Both herds ranged the same woods, and fed upon the same herbage. It is presumable, therefore, that the disorder was produced by the water; and in confirmation of this opinion, this individual farther stated to me, that suspecting a spring at which his cattle drank to be the origin of the evil, he set to work felling trees around it, so as to exclude his stock from it, and that afterwards they suffered no more with the disease for several years. At length, however, it occurred again, and on examination it was found, that the spring had become accessible from the decay of the timber. The enclosure being repaired, and the cattle shut out from the water, the disease a second time disappeared. Another farmer of respectability told me that a number of his sheep, kept upon a grass lot on account of wolves, had died with the symptoms of milk-sickness. During every season several had died for a series of years, until his flock was nearly destroyed. He at last began to suspect a dripping spring in the *lot*, as the source of the mischief, and accordingly took measures to secure the animals against it, whereupon the disease ceased. In this *lot* it is not probable that there existed any vegetable to which the affection could be attributed."

*Prevention of the Disease.* "That clover in a green state will, to a great extent, obviate the disease in cattle feeding freely upon it, is a fact not questioned by those who live in milk-sick regions. Other nutritious, succulent vegetables may act in a similar manner, but their efficacy is not so well established. The beneficial effects of clover are shown, by sowing it in ground known to have afforded the milk-sick poison; in which case the disease ceases to appear, especially in cows yielding milk. This experiment has been repeatedly performed in Tennessee and Indiana, and has generally been attended with success. Fields known to have contained the cause of the disorder, have been freed from it in repeated instances, by setting it well in clover; and it has again appeared on the clover being ploughed up, and the field sowed in grain. Thus this plant would appear to possess the power, in some way, of correcting the poison of milk-sickness."

*Treatment.*—"In the incipient stage of this disease," says Dr. White, "rest, and some aperient medicine will generally ward it off, and restore the patient in a few days to health. For this purpose he has found the sulphate of magnesia superior to all other remedies. Some cases treated by this article alone, where nausea and vomiting had come on, have been relieved. The vomiting, when it supervenes, is best treated by chamomile tea, which allays gastric irritation. After the bowels have been evacuated freely, the patient is considered convalescent, and nothing more is necessary than to keep them soluble. This is best done by the medicine already recommended. Since we adopted this practice, we have not lost a single patient out of some eight or ten."

Dr. Shelton uses emetics and cathartics, and bleeds when the vascular action is high. He remarks, "The most certain and efficacious emetic that can be given, is the euphorbia ipecacuanha in tincture; three ounces of the bruised root are infused in a pint of diluted spirits of wine, of which one or two table-spoonfuls are given every ten minutes until free emesis is induced. The superiority of this medicine over the foreign article seems to consist chiefly in its superior purgative powers, an effect so necessary in this disease. When free vomiting can be thus speedily produced, little else, in most cases, will be requisite than to follow it with a single dose or two of calomel. In more protracted cases the use of mercurial cathartics will be necessary for a longer time."

*The Transylvania Journal of Medicine, March,* 1836.

### *On certain Pathological Changes in the Arterial System.* By M. Bizot. (*Thesis.*)

The memoir of M. Bizot on this subject was composed under the auspices of M. Louis, and is the result of the examination of about 160 hearts and arterial systems. The following are the principal conclusions arrived at:—The red colour of the internal membrane of the arteries, without other change, must not be considered as morbid. The alterations which were found are divided into those whose progress was primarily acute, and those primarily chronic. Of the former there is but one, which is an exudation more or less thick, of the appearance and consistence of albumen, rosy or transparent, without colour, and very adherent to the inner membrane. From tracing the transformation of this exudation, M. Bizot believes that the cartilaginous patches have commenced in this manner, and he rejects the opinion of M. Andral, that these patches are developed between the inner and middle membrane. When this albuminous exudation is secreted in isolated patches, its formation does not appear to produce general symptoms; but, if it attacks a large surface, (as the whole aorta, for instance,) formidable symptoms appear, as the author has observed in three cases which he has reported. In these three cases the symptoms were similar: œdema accompanied with febrile disturbance, without symptoms which could be referred to the heart, or the principal organs essential to life; and, on examination, the same lesion was discovered in all, a false membrane lining the whole inner surface of the aorta. This disease is very different from acute aortitis described by MM. Bertin and Bouillaud. The changes of a chronic kind are either common to the whole arterial system, or only affect the arteries of the limbs. The first commences by small, yellowish points, which, developed between the inner and middle membrane, may experience many transformations. According to M. Bizot, the changes described by authors under the names of spots, pustules, abscesses, atheroma, steatoma, &c., have all the same origin, which is a little almost imperceptible spot developed without any trace of inflammation between the most internal coats of the artery, and terminating alike in ulceration. The spots may also be transformed into bony matter, but without passing through the state of cartilage. In the arteries of the limbs, ossification appears to take place in the substance of the middle coat. *Archives Gen. de Med., Juillet*, 1836.

---

### *Pathological Observations.* By J. F. H. Albers, m.d., of Bonn.

I. *Observations on the Par Vagum.* In forty-three cases of hooping-cough, which terminated fatally in the first stage, the par vagum exhibited nothing remarkable from its origin to its entrance into the œsophagus. On the other hand, in four individuals of a full and scrofulous habit, the left nerve was found in one, and the right nerve in three instances, slightly reddened externally. This redness, which was always on the side on which the body had lain, was of the same description as that observed in the par vagum of plethoric subjects who had died of fever. In seven cases of fever with intestinal ulceration, (*Dothinenteritis,*) the right nervus vagus was twice, the left once, found reddened from infiltration of blood into the sheath of the nerve. The redness, however, disappeared after the nerve had been steeped in water for some time.

A robust young man, aged twenty-seven, was, on the 14th of July, attacked with violent febrile symptoms; in the evening, difficulty in breathing, anxiety, convulsions, and ultimately delirium, ensued; under which symptoms he died about midnight. On opening the body, the cervical portion of the left nervus vagus was found reddened and softer than usual. Being kept for some hours in cold water, it gradually lost its redness, and assumed a yellowish-white appearance.*

* It is observed, that when plethoric subjects have lain for some time with the head much raised, so that the cervical portion is exposed to pressure, the nerve is frequently found deeply tinged with red, owing merely to cadaverous congestion. In such cases, however, the blood is readily extracted by water, and the natural colour of the nerve returns; which, however, did not happen in the above case.

In fifteen persons who had died of tubercular phthisis, both branches of the nerve appeared unusually strongly developed, and it was remarkable that the right nerve was far more so than the left. In ulcers of the œsophagus and of the trachea, the nerve was found to be entirely obliterated by the ulceration. Tilgen describes a remarkable instance of a species of tumour exhibited by the nervus vagus. A fungus medullaris had formed in the mediastinum, and, by pressure, had disturbed the functions of the trachea and the œsophagus.

II. *Tumours in the Cerebellum.* The observations of Billard, respecting a difference in the crying of children in diseases of the brain, have hitherto received no confirmation from others. In two cases described by the author, the tone, which was only uttered during inspiration, was extraordinarily fine and interrupted, and, in fact, so striking, that in the second case cerebral disease was immediately recognized by the cry alone. In the first case, the symptoms gave rise to suspicion of inflammatory irritation at the base of the brain; in the second, convulsions were the predominant symptoms. Swellings, the size of a nut, were found in one of the hemispheres of the cerebellum, accompanied by a great quantity of serous fluid in the neighbourhood of the cerebellum, the medulla oblongata and spinal marrow.

The change of voice was probably caused by the serous fluid collected about the medulla, as, by that means, injury would accrue to the respiratory nerves, the vagus, glosso-pharyngeus, the accessorius Willisii, and the facialis, which exert a powerful influence over the complicated mechanism of respiration, and consecutively on the brain.

III. *Case of divided Uterus.* The individual, aged forty-six, of a weakly constitution, had never enjoyed good health, and had never had the catamenial discharge: from the time of puberty, however, she had almost every month been seized with lancinating pains in the left hypochondrium, accompanied by vomiting and other pains, which seemed to bear down towards the lower part of the pelvis. In her 26th year, during a violent febrile attack, a slight hemorrhage once occurred from the vagina. At the age of forty-six, she had recourse to medical advice for the pain in the left hypochondrium, which was relieved by a mild antiphlogistic treatment. On dismissing the patient, the author examined the genital parts, and found the external ones in a natural state; the vagina, however, was only an inch in length, and ended in a blind sack. A year afterwards, the patient died of peritonitis.

On examining the internal genital organs, the author found the vagina as above described. About an inch and a half laterally from it, two oblong bodies were discovered, of the size of a walnut; but not manifesting any actual connexion with the vagina. Laterally, from the upper part of each, a bundle of fibres extended towards another body, which was suspected to be the malformed or degenerated ovarium. The two oblong bodies, which must be regarded as the rudiments of the uterus, contained each a cavity corresponding to their outer forms, and lined with a species of mucous membrane. The substance of the parietes was white, fibrous, and about two lines in thickness; there were no distinct traces of the round ligaments, or of the alæ vespertilionis, these being altogether lost in the cellular masses in which the above parts lay imbedded.

The Medical Association of Toulouse has recorded a similar instance of sacciform vagina, and of probably deficient uterus. Another case is related by Dr. Hohlfeld, of Berlin, of a woman, aged twenty-five, in whom an incision was unsuccessfully made through the imperforated extremity of the vagina.

*Kleinert's Repertorium*, 1836.

---

### *On the Effect of repeated Loss of Blood on the Constitution of the Blood-vessels.* By Dr. Hermann Nasse.

We have not yet had an opportunity of seeing the original work of Dr. Nasse on the physiology and pathology of the blood, which he has just published, and in which he has consigned the result of observations and experiments carried on by him for no less a period than ten years. The following are some of the principal results ascertained respecting the influence of loss of blood on the state of the fluid, as recorded by Dr. Albers in the Hanoverian Annals.

1. The blood flows from the vein with less force and in a smaller stream.
2. Its temperature is diminished.
3. The blood cools more rapidly in the first minutes.
4. Its colour is brighter.
5. Its specific gravity is diminished.
6. The coagulation takes place sooner.
7. The serum separates frequently more speedily from the clot.
8. The disposition to form the fibrinous coat is increased.
9. The quantity of water is increased.
10. The coagulum is less firm.
11. The specific gravity of the serum is diminished.
12. The powder-like sediment is augmented.
13. The height of the remaining cruor in "whipped" blood is lessened.
14. The blood putrefies more speedily.
15. The fibrinous substance frequently increases, but becomes tenderer, and is more readily decomposed.
16. The quantity of globules is diminished.
17. The quantity of iron is diminished.
18. The albuminous portion of the blood increases somewhat.
19. The quantity of fat in the serum and in the fibrine is increased.

*Hannoversche Annalen für die gesammte Heilkunde.* B. 1, Heft. 3. 1836.

---

*Communication of Spinal Abscess with the Bronchi.* By Dr. Stannius.

Eichenbrod, aged thirty-three years, about two years ago, after exposure to cold, suffered dragging pains in the back and sides of the chest, frequent feeble rigors, and an almost constant dry short cough. He was harassed by constant sweatings. The pains gradually increased, and the movement of his body became difficult. After very violent pains in the back, two small and not very painful boils formed, which were opened some months before the patient entered the hospital, (October, 1833.) A considerable quantity of pus escaped, and relieved the patient. The openings diminished, but never closed, and there was a daily discharge from them of a purulent fluid. When first seen by Dr. Stannius, the patient was emaciated, troubled with a constant clammy sweat; feverish, particularly towards evening. A cough caused him great pain in the back and sides of the thorax: every movement was laborious. Between the scapula and spine were two small round openings, with somewhat swollen borders. A probe could be passed for several inches, upwards, downwards, and forwards, showing that several sinuses were connected with these openings. A moderately clear and not very consistent fluid escaped from them, in small quantity; sometimes, particularly after violent cough, a large stream of purulent matter poured forth. The thorax was narrow and small; the sound on percussion dull. There was at first but scanty expectoration with the cough; but subsequently it was abundant, and of a muco-purulent character; expectorated without difficulty. Whilst the evacuation by the mouth continued, that by the fistulous openings almost entirely ceased. The expectoration by the mouth became suddenly impeded; the anxiety of the patient and the dyspnœa increased; a violent pain was complained of in the left side of the thorax, and death took place in four days from the date of the cessation of expectoration.

After death were found strong adhesions of the pleuræ of the left side, as well as of both lungs to the diaphragm. Both lungs adhered closely to the back, as did the upper lobe of the right lung, laterally and anteriorly. On the outer and back surface of the right lung was a thick layer of a matter, which was partly solid and almost cartilaginous, and partly cheesy; and which, although in smaller quantity, was found on the posterior surface of the left lung. When the lungs were taken out, on the side of the upper lobe of the right lung were seen two small circular openings, about the circumference of a pea. On its posterior surface were six similar apertures. In their vicinity was a large quantity of the same adhesive matter; which united the opposite surface of the pleuræ in other parts. These openings communicated with the bronchial ramifications. There was one fistulous aperture on the posterior surface of the upper lobe of the left lung. All the bodies and transverse processes of the vertebra corresponding to the thoracic cavity were, together with the ribs, particularly of the right side, affected with caries. From the two external openings, passing between the tendinous and muscular layers of the back, proceeded fistulæ in various directions, and increasing in number as they approached nearer the spine. These fistulæ were lined by a fine membrane, which was almost entirely covered by a closely adherent, yellowish-brown, clay-coloured, semifluid substance.

The pus which was formed in consequence of the disease of the spine and ribs was then evacuated in two directions; externally, through the openings in the back, and internally into the cavities of the pleuræ, and through the bronchial ramifications. *Wochenscrift für die Gesammte Heilkunde.* No. 8. 1836.

